# Treatment Paradigms for Patients with Metastatic Non-Small Cell Lung Cancer, Squamous Lung Cancer: First, Second, and Third-Line

**DOI:** 10.3389/fonc.2014.00157

**Published:** 2014-06-27

**Authors:** Abdulaziz Al-Farsi, Peter Michael Ellis

**Affiliations:** ^1^Department of Oncology, Juravinski Cancer Centre, McMaster University, Hamilton, ON, Canada

**Keywords:** non-small cell lung cancer, squamous cancer, molecular abnormalities, chemotherapy, EGFR inhibitors, immunotherapy, novel agents

## Abstract

Historically, the treatment algorithm applied to non-small cell lung cancer (NSCLC) was the same for all histologic subtypes. However, recent advances in our understanding of the molecular profiles of squamous and non-squamous NSCLC have changed this perspective. Histologic subtype and the presence of specific molecular abnormalities have predictive value for response to and toxicity from therapy, as well as overall survival. For patients with squamous NSCLC, a platinum agent plus gemcitabine, or paclitaxel is recommended as first-line therapy. The role of epidermal growth factor receptor monoclonal antibodies is uncertain. Maintenance therapy is not widely recommended, although data exist for the use of erlotinib. The standard recommendation for second-line therapy is docetaxel and erlotinib should be considered as second or third-line therapy. There is ongoing research identifying molecular targets in squamous NSCLC and many agents are in early phase clinical trials. Immunotherapeutic approaches targeting programed death-1 receptor and its ligand (PD-L1) appear promising.

## Introduction

Historically, one simplified management algorithm was applied to all patients with non-small cell lung cancer (NSCLC). A platinum agent combined with paclitaxel, docetaxel, vinorelbine, or gemcitabine was recommended for first-line chemotherapy (1, 2). Second-line chemotherapy at the time of disease progression could include docetaxel (3, 4), or pemetrexed (5) and erlotinib was recommended as second/third-line therapy (6). There was no evidence that histologic subtype of NSCLC impacted on the response to, or survival gained from chemotherapy (7).

However, important differences exist between the major subtypes of NSCLC, which have prognostic and predictive value. There is evidence from some trials, that patients with squamous histology have worse overall survival (OS) than patients with adenocarcinoma (8). Additionally, qualitative interactions exist between histology and the efficacy of some treatments (9). Differences also exist in the molecular profile of squamous and non-squamous NSCLC (10). Mutations of the *Epidermal Growth Factor Receptor* (*EGFR*), or *Kirsten Rat Sarcoma* (*K-RAS*) genes are rare in squamous cancers (10). However, EGFR protein overexpression, or increased gene copy number occur commonly. Differences exist in the expression of thymidylate synthase (TS) (11). Higher messenger RNA (mRNA) and protein levels for TS are seen in squamous cancers compared to non-squamous cancers, although there is no direct clinical correlation between levels of TS mRNA and measures of response to treatment. Nevertheless, separate treatment algorithms for squamous cancers have evolved. In this review, we summarize the approach to the management of squamous NSCLC.

## First-Line Therapy for Squamous Cancers

Randomized trials in the 1980s demonstrated that chemotherapy improved OS as well as quality of life (12). These initial studies of platinum-based chemotherapy did not observe any differential response, or survival, based on histologic subtype. Multiple subsequent studies comparing various platinum-based chemotherapy regimens demonstrated similar response rates and OS (1, 2, 13). A retrospective review by the Southwest Oncology Group (SWOG), of systemic therapy trials of anti-microtubule agents, also showed no differential effect in outcomes according to histologic subtype (7).

More recent data demonstrate that histologic subtype is predictive of a differential response rate, OS, or toxicity profile from certain systemic therapies (9, 14, 15). The JMDB trial randomized 1725 patients with NSCLC (all histologic subtypes), to six cycles of first-line chemotherapy with cisplatin and gemcitabine, or cisplatin and pemetrexed (16). The primary outcome for the trial showed that cisplatin and pemetrexed was non-inferior to cisplatin and gemcitabine (median OS 10.3 months for both arms, HR 0.94, 95% CI 0.84–1.05). A planned sub-group analysis demonstrated evidence of a qualitative interaction between treatment effect and histology (interaction *p* = 0.0011). OS was significantly improved for patients with non-squamous histology randomized to cisplatin and pemetrexed, versus cisplatin and gemcitabine (11.8 vs. 10.4 months, HR 0.81, 95% CI 0.70–0.94). However, in patients with squamous cancer, OS was significantly worse for patients randomized to cisplatin and pemetrexed (9.4 vs. 10.8 months, HR 1.23, 95% CI 1.0–1.53).

Similar evidence of an interaction between histology and treatment efficacy for pemetrexed was observed in the JMEN trial evaluating maintenance therapy with pemetrexed, following four cycles of platinum-based chemotherapy (17). The improvement in progression free survival (PFS) from maintenance pemetrexed was significantly greater in patients with non-squamous histology than squamous histology (HR 0.44, 95% CI 0.36–0.55 vs. HR 0.69, 95% CI 0.49–0.98; interaction *p* = 0.036). Similarly the observed improvement in OS was limited to patients with non-squamous histology (HR 0.70, 95% CI 0.56–0.88 vs. HR 1.07, 95% CI 0.77–1.50, interaction *p* = 0.033). A second large randomized trial of maintenance therapy following first-line platinum-based chemotherapy evaluated erlotinib (18). The SATURN trial, which included approximately 40% of patients with squamous cancers, showed a modest improvement in OS for patients randomized to maintenance erlotinib compared with placebo (12 vs. 11 months, HR 0.81, 95% CI 0.70–0.95). There was no evidence of any interaction between treatment and histology (squamous HR 0.86, 95% CI 0.68–1.10, adenocarcinoma HR 0.77, 95% CI 0.61–0.97).

Several trials have evaluated gemcitabine as maintenance therapy (19–21). Brodowicz et al. (20) randomized patients to maintenance gemcitabine or placebo, after four cycles of cisplatin and gemcitabine. There was a significant improvement in TTP, but no significant improvement in OS. In contrast, Belani et al. (19) found no improvement in PFS or OS, for continuation maintenance gemcitabine. Data were not analyzed according to histology. Lastly, the IFCT-0502 trial randomized patients to gemcitabine, erlotinib, or observation, after first-line cisplatin and gemcitabine (21). Improvements in PFS were observed for patients randomized to both gemcitabine and erlotinib, compared with observation, but no differences were observed in OS. For patients randomized to gemcitabine, the improvement in PFS seemed less for patients with adenocarcinoma than other histologies (HR 0.98 vs. 0.79). There is insufficient data to recommend maintenance gemcitabine in this setting.

The selection of first-line therapy for NSCLC is also influenced by differing toxicity profiles between the histologic subtypes. Major hemoptysis was observed in the randomized phase II trial evaluating the addition of bevacizumab to first-line carboplatin and paclitaxel (14). There was an excess risk of life threatening hemoptysis in patients with squamous cancers and these patients were excluded from subsequent trials of bevacizumab in NSCLC. More recently, an open-label single-arm phase II study, the BRIDGE trial, evaluated delaying bevacizumab until after the third cycle of carboplatin and paclitaxel, in 31 patients with squamous NSCLC (22). Severe pulmonary hemorrhage was observed in 1 patient and grade 3 toxicities occurred in 9 of 31 patients overall. The authors conclude that bevacizumab remains experimental therapy in patients with squamous NSCLC.

Additional trials evaluating other targeted agents have also shown worse outcomes for patients with squamous NSCLC. The ESCAPE trial randomized 926 patients with advanced NSCLC (all histologies) to carboplatin, paclitaxel with or without sorafenib (15). The study was discontinued early following an interim analysis demonstrating futility. In the final analysis of this trial, patients with squamous histology (*n* = 223) randomized to chemotherapy plus sorafenib had worse OS than patients randomized to chemotherapy alone (8.9 vs. 13.6 months, 95% CI 1.22–2.81), as well as lower PFS (4.3 vs. 5.8 months, HR 1.31 95% CI 0.94–1.83).

However, for some agents, squamous histology appears to predict better outcomes from treatment. The BMS-099 phase III trial randomized patients with advanced NSCLC to carboplatin and paclitaxel or docetaxel, with or without cetuximab (23). Although the response rate was better for patients randomized to chemotherapy plus cetuximab (25.7 vs. 17.2%, *p* = 0.007), there was no difference in PFS, or OS. There was a trend to greater benefit from the addition of cetuximab to chemotherapy, in patients with squamous NSCLC (HR 0.7, 95% CI 0.47–1.05) compared to patients with adenocarcinoma (HR 0.9, 95% CI 0.71–1.14). A second phase III trial, the FLEX trial, evaluated the addition of cetuximab to cisplatin and vinorelbine chemotherapy in patients with advanced NSCLC demonstrating EGFR expression on immunohistochemistry (IHC) (24). There was a modest improvement in OS for patients randomized to chemotherapy plus cetuximab compared with chemotherapy alone (11.3 vs. 10.1 months, HR 0.871, 95% CI 0.762–0.996). There was a trend for greater benefit in patients with squamous histology (HR 0.8, 95% CI 0.64–1.0) compared to patients with adenocarcinoma (HR 0.94, 95% CI 0.77–1.15). However, a meta-analysis of four trials of first-line cetuximab plus chemotherapy in advanced NSCLC did not find clear evidence for a differential effect of treatment according to histology (25).

There does appear to be a differential effect of treatment according to histology for necitumumab, a second EGFR monoclonal antibody. The INSPIRE trial evaluating the addition of necitumumab to cisplatin and pemetrexed in non-squamous NSCLC patients, was stopped early because of an increased risk of thromboembolic complications in the necitumumab arm (26). However, a press release from Eli Lilly earlier in 2014, announced that a similar trial in patients with squamous NSCLC, had demonstrated improved OS for patients receiving cisplatin plus gemcitabine in combination with necitumumab, compared with cisplatin and gemcitabine alone. These data should be available during 2014.

The available data support adopting a different algorithm for first-line therapy of patients with squamous NSCLC compared with non-squamous histology. While cisplatin and pemetrexed appears to be the preferred chemotherapy in patients with non-squamous NSCLC, pemetrexed appears to be an ineffective drug in patients with squamous cancers. The JMBD trial did show that cisplatin and gemcitabine was superior chemotherapy in patients with squamous cancers (16). However, other trials, such as ECOG 1594, have demonstrated similar survival to cisplatin and gemcitabine from alternate chemotherapy regimens such as carboplatin and paclitaxel (2). Either one of these regimens would be an appropriate choice for first-line chemotherapy in patients with advanced squamous NSCLC. The role of EGFR monoclonal antibodies is somewhat uncertain. The benefit from cetuximab is very modest and there are additional toxicities. Therefore, it has not been widely implemented into first-line treatments. Data on necitumumab will need to be examined more closely once it is presented, in order to determine whether it should be incorporated into routine management of patients with squamous NSCLC. Finally, maintenance options in squamous NSCLC are limited. Pemetrexed is ineffective as maintenance therapy for patients with squamous cancers and the benefit of erlotinib is modest. Therefore, maintenance therapy in this population is not routinely employed.

## Second-Line Chemotherapy for Squamous Cancers

Second-line chemotherapy for NSCLC has been widely adopted since publication of two randomized trials in 2000 (3, 4). The TAX 317 trial randomized patients to docetaxel or best supportive care following first-line platinum chemotherapy (4), whereas the TAX 320 trial randomized patients to docetaxel vs. either vinorelbine, or ifosfamide (3). Both trials showed modest, but significant improvements in OS and the TAX 317 trial also demonstrated improvement in lung cancer related symptoms (27). A further trial compared second-line therapy with docetaxel to pemetrexed (JMEI) (5). This trial demonstrated that pemetrexed was non-inferior to docetaxel (median OS 8.3 vs. 7.9 months, HR 0.99, 95% CI 0.83–1.2). Secondary outcomes including response rate, time to progression, and duration of response were also similar between the two groups. Therefore, pemetrexed and docetaxel were both established as options for second-line chemotherapy for NSCLC.

A retrospective analysis was subsequently undertaken of the JMEI trial to look for an interaction between treatment effect and histology (8, 9). Similar to data in the first-line setting, there was a qualitative interaction between histology and treatment effect. Patients with non-squamous histology treated with pemetrexed, had significantly longer survival (9.3 vs. 8.0 m, HR 0.78 95% CI 0.60–1.02, interaction *p* = 0.04). On the other hand, patients with squamous histology who received pemetrexed had inferior survival (6.2 vs. 7.4 months, HR 1.56 95% CI 1.08–2.26). This analysis primarily showed that patients with squamous NSCLC treated with pemetrexed had an inferior outcome.

These data support a differential approach to second-line chemotherapy according to histology. Docetaxel is recommended as second-line chemotherapy in patients with squamous type NSCLC, whereas pemetrexed is recommended for patients with non-squamous NSCLC. However, due to the adoption of pemetrexed in the first-line setting, docetaxel remains an option for second-line chemotherapy, in patients with non-squamous histology.

## Erlotinib Therapy in Patients with Squamous Cancers

Many patients with advanced NSCLC are still candidates for further systemic therapy, at the time of progression on second-line chemotherapy. There is little evidence though, to support the use of a third-line of chemotherapy. However, the results of the BR21 trial of erlotinib vs. best supportive care support the use of erlotinib as second or third-line therapy (6). This trial enrolled 731 patients, including patients with poor performance status (ECOG 2 and 3), who had previously received one or two lines of chemotherapy (49% received two prior lines of chemotherapy). The trial demonstrated that erlotinib significantly improved PFS (2.2 vs. 1.8 months, HR 0.61, 95% CI 0.51–0.74) and OS (6.7 vs. 4.7 months, HR 0.70, 95% CI 0.58–0.85). In addition, time to deterioration in cough, dyspnea, and pain were all significantly improved for patients randomized to erlotinib. The benefit of erlotinib was similar in both second and third-line settings.

Since publication of the BR21 trial, there has been a considerable body of evidence demonstrating that the presence of activating mutations of the *EGFR* gene are strongly predictive of benefit from an EGFR tyrosine kinase inhibitor (TKI) (28). It has been argued that the benefit of an EGFR TKI is limited to such patients with an *EGFR* mutation. Given that *EGFR* mutations are rare in patients with squamous cancers, it has also been argued that EGFR TKIs are ineffective in this group of patients as well. However, the data from both BR21 and the SATURN trial of maintenance erlotinib (18), demonstrate that patients who are *EGFR* wild type also benefit from EGFR TKIs, although the magnitude of benefit may be smaller. Similarly, available data do not support the contention that patients with squamous cancers fail to benefit from an EGFR TKI. In the BR21 trial, 31% of patients had squamous histology. The magnitude of benefit from erlotinib appeared similar in patients with squamous cancers (HR 0.8, 95% CI 0.6–1.0) and patients with adenocarcinoma (HR 0.7, 95% CI 0.6–0.9). Therefore, erlotinib should be considered as a second or third-line option for treatment in patients with squamous NSCLC. To date no other agents have be shown to improve survival in this setting.

## Future Directions

The molecular profile of adenocarcinomas of the lung has been well described (29). Common mutations in lung adenocarcinoma include *K-RAS*, *EGFR*, as well as translocations of the *Anaplastic Lymphoma Kinase (ALK)* gene. However, there has been less research characterizing molecular abnormalities in lung cancer patients with squamous tumors. The cancer genome atlas (TCGA) network published data on genomic and epigenetic analysis of 178 squamous NSCLC patients from across the world (30). The data suggest squamous NSCLC’s are genetically complex tumors. A high proportion of squamous cancers (171 of 178) contain one or more mutations in tyrosine kinases, serine/threonine kinases, PI3K catalytic and regulatory subunits, nuclear hormone receptors, G protein-coupled receptors, proteases, and tyrosine phosphatases. Common molecular abnormalities include mutations and amplification of *fibroblast growth factor receptor gene* (*FGFR*), mutations of *phosphatidylinositol 3-kinase*, *catalytic subunit alpha gene (PIK3CA)*, *phosphatase and tensin homolog gene (PTEN)*, *discoid domain receptor 2 gene* (*DDR2*), *BRAF*, as well as *EGFR* amplification (31). This would suggest that squamous cancers have a different molecular phenotype, hence the need for a separate treatment algorithm. Multiple clinical trials are ongoing evaluating agents targeting these molecular abnormalities, which will further refine the treatment algorithm in squamous NSCLC.

Immune based therapies also appear to be gaining momentum in NSCLC. There is considerable interest in immune checkpoint regulation in NSCLC. In particular, therapy directed toward the programed death-1 receptor (PD-1), or its ligand PD-L1 has shown considerable promise in early phase clinical trials. Phase I trials of several PD-1 receptor monoclonal antibodies, including nivolumab (32), MK-3475 (33), as well as the PD-L1 antibody MPDL-3280A (34) have all shown evidence of anti-tumor activity in heavily treated NSCLC patients. Approximately 20% of patients have shown objective tumor responses and many of these responses have been durable beyond 1 year. Response rates of 60–70% were observed in patients with tumors expressing PD-L1, although there is a need to develop valid and reliable methods of assessment of this. Of note, the response rates among patients with squamous cancers appear to be higher than response rates in patients with adenocarcinoma, although it is unclear whether histology or some underlying molecular difference may account for these findings. Data also exist for the CTLA-4 antibody, ipilimumab. A randomized phase II trial of chemotherapy with six cycles of carboplatin and paclitaxel, chemotherapy plus ipilimumab in cycles one to four (concurrent) and chemotherapy plus ipilimumab in cycles three to six (phased). Patients randomized to carboplatin, paclitaxel, and phased ipilimumab had improved PFS compared with the control arm (HR 0.72, 95% CI 0.50–1.06, *p* = 0.05). These data require confirmation in phase III trials. The data suggest patients with squamous cancers may have more benefit from the addition of ipilimumab (HR 0.55, 95% CI 0.27–1.12) than patients with non-squamous cancers (HR 0.81, 95% CI 0.53–1.26).

## Conclusion

It is clear that a different treatment algorithm has emerged for patients with squamous NSCLC. In the first-line setting platinum doublets involving gemcitabine, or potentially paclitaxel should be considered. The role of EGFR monoclonal antibodies such as cetuximab and necitumumab, require further clarification. Treatment options beyond progression of first-line therapy include docetaxel and erlotinib. Multiple trials are currently examining new therapies targeting the common molecular abnormalities observed in squamous lung cancers.

## Conflict of Interest Statement

Peter Michael Ellis has received honoraria from Eli Lilly and Roche for advisory board meetings. The other co-author reports no conflicts of interest.
